# Visceral Leishmaniasis and HIV Co-infection in Bihar, India: Long-term Effectiveness and Treatment Outcomes with Liposomal Amphotericin B (AmBisome)

**DOI:** 10.1371/journal.pntd.0003053

**Published:** 2014-08-07

**Authors:** Sakib Burza, Raman Mahajan, Prabhat K. Sinha, Johan van Griensven, Krishna Pandey, María Angeles Lima, Marta Gonzalez Sanz, Temmy Sunyoto, Sunil Kumar, Gaurab Mitra, Ranjeet Kumar, Neena Verma, Pradeep Das

**Affiliations:** 1 Médecins Sans Frontières, New Delhi, India; 2 Institute of Tropical Medicine, Antwerp, Belgium; 3 Rajendra Memorial Research Institute of Medical Sciences, Patna, Bihar, India; 4 Médecins Sans Frontières, Barcelona, Spain; 5 Sri Krishna Medical College and Hospital, Muzaffarpur, Bihar, India; The Ohio State University, United States of America

## Abstract

**Background:**

Visceral Leishmaniasis (VL; also known as kala-azar) is an ultimately fatal disease endemic in the Indian state of Bihar, while HIV/AIDS is an emerging disease in this region. A 2011 observational cohort study conducted in Bihar involving 55 VL/HIV co-infected patients treated with 20–25 mg/kg intravenous liposomal amphotericin B (AmBisome) estimated an 85.5% probability of survival and a 26.5% probability of VL relapse within 2 years. Here we report the long-term field outcomes of a larger cohort of co-infected patients treated with this regimen between 2007 and 2012.

**Methods and Principal Findings:**

Intravenous AmBisome (20–25 mg/kg) was administered to 159 VL/HIV co-infected patients (both primary infections and relapses) in four or five doses of 5 mg/kg over 4–10 days. Initial cure of VL at discharge was defined as improved symptoms, cessation of fever, improvement of appetite and recession of spleen enlargement. Test of cure was not routinely performed. Antiretroviral treatment (ART) was initiated in 23 (14.5%), 39 (24.5%) and 61 (38.4%) before, during and after admission respectively. Initial cure was achieved in all discharged patients. A total of 36 patients died during follow-up, including six who died shortly after admission. Death occurred at a median of 11 weeks (IQR 4–51) after starting VL treatment. Estimated mortality risk was 14.3% at six months, 22.4% at two years and 29.7% at four years after treatment. Among the 153 patients discharged from the hospital, 26 cases of VL relapse were diagnosed during follow-up, occurring at a median of 10 months (IQR 7–14) after discharge. After accounting for competing risks, the estimated risk of relapse was 16.1% at one year, 20.4% at two years and 25.9% at four years. Low hemoglobin level and concurrent infection with tuberculosis were independent risk factors for mortality, while ART initiated shortly after admission for VL treatment was associated with a 64–66% reduced risk of mortality and 75% reduced risk of relapse.

**Significance:**

This is the largest cohort of HIV-VL co-infected patients reported from the Indian subcontinent. Even after initial cure following treatment with AmBisome, these patients appear to have much higher rates of VL relapse and mortality than patients not known to be HIV-positive, although relapse rates appear to stabilize after 2 years. These results extend the earlier findings that co-infected patients are at increased risk of death and require a multidisciplinary approach for long-term management.

## Author Summary

Fifty percent of all visceral leishmaniasis (VL) cases globally occur in India, where up to 90% of cases occur in the state of Bihar. There are also an estimated 300,000 people in Bihar living with HIV/AIDS. Patients with HIV who are treated for VL typically have much worse outcomes than VL patients who are HIV-negative, yet there exists very little evidence suggesting more effective treatments for this group. Between 2007–2012, with support of the Rajendra Memorial Research Institute (RMRI), Médecins Sans Frontières (MSF) treated 8,749 VL patients in Bihar using liposomal amphotericin-B (AmBisome). Here we describe the characteristics and long-term outcomes of a subgroup of 159 HIV-VL co-infected patients treated within this program over the 5-year period. Their estimated mortality risk was 14.3% at six months after treatment, 22.4% at two years and 29.7% at four years. Estimated risk of relapse was 16.1% at one year, 20.4% at two years and 25.9% at four years. We conclude that treatment of HIV-VL co-infected patients with 20–25 mg/kg of liposomal amphotericin-B is well tolerated and relatively effective. However, HIV-VL co-infection is a complex chronic disease with high early mortality and much worse outcomes than VL alone, and requires a multidisciplinary long-term management strategy.

## Introduction

One third of all HIV patients worldwide live in regions where leishmaniasis is endemic [1]. Visceral leishmaniasis (VL) caused by the parasite *L. donovani* is endemic to Bihar, a populous state of 110 million people in East India, which carries an estimated 40% of the world's VL burden [2]. Although Bihar has a relatively low prevalence of HIV (between 0.22–0.33%), its high population density means that in absolute numbers an estimated 300,000 people in the state live with HIV/AIDS [3].

Moreover, Bihar is one of the few states in India where the rate of new HIV infections is increasing [4]. This has major implications for VL co-infection: like other opportunistic infections in HIV patients, *Leishmania* amastigotes have evolved strategies to survive and multiply within macrophages [5], which are enhanced by HIV co-infection [6] and accelerate progression of disease [7]. This may help explain why the risk of developing VL is estimated to be between 100 and 2300 times higher in HIV-infected individuals than in those who are HIV-negative. [8]. Data on the prevalence of HIV-VL co-infection in India is scarce, although estimates range from 2–5.6% [9]–[14]. HIV-VL co-infection therefore appears to be a growing public health issue in India.

Yet the evidence base regarding best treatment practices for co-infected patients is limited, due to a lack of randomized controlled trials and to the fact that most available data comes from observational studies with relatively short follow-up periods and often with high rates of loss to follow-up [15]. Nevertheless, worse outcomes in almost every respect have consistently been reported in this patient group when compared to patients not known to be HIV-positive—for example, in terms of higher relapse rates, mortality, and VL drug toxicity and resistance [15].

Currently the Indian treatment guidelines for VL do not differentiate treatment of HIV-VL co-infected patients from that of other patients presenting with VL. First-line treatment for all VL patients in India is 28 days of oral miltefosine (where not contra-indicated), although the government is currently assessing the use of single-dose AmBisome and lower-dose combinations therapies [16] as recommended by the World Health Organization (WHO) [17]. However, India has not developed a contingency plan for HIV-VL patients.

Since 2007, Médecins Sans Frontières (MSF) has collaborated with the Rajendra Memorial Research Institute (RMRI) and the National Vector Borne Disease Control Program (NVBDCP) to implement a VL treatment program within Ministry of Health (MoH) facilities in Vaishali district, one of the most highly endemic areas for VL in Bihar, The program has treated over 8,500 patients using 20 mg/kg liposomal amphotericin B (AmBisome, Gilead Pharmaceuticals, Foster City, CA, USA). High-dose liposomal amphotericin B is currently recommended by WHO for first-line treatment of HIV-VL co-infection [17]. We have exclusively used AmBisome, a brand name for liposomal amphotericin B, since it is the only preparation of this medication that has received stringent regulatory approval for use in VL [16].

In this retrospective observational cohort study of routinely collected program data, we describe the baseline characteristics of the 159 HIV-VL co-infected patients treated with liposomal amphotericin B in the Bihar program between July 2007 and August 2012. We then describe the outcomes for VL immediately after treatment and in the longer term (up to 5 years), the latter being crucial to monitor given the chronic nature of HIV infection.

## Methods

**Figure 1:**
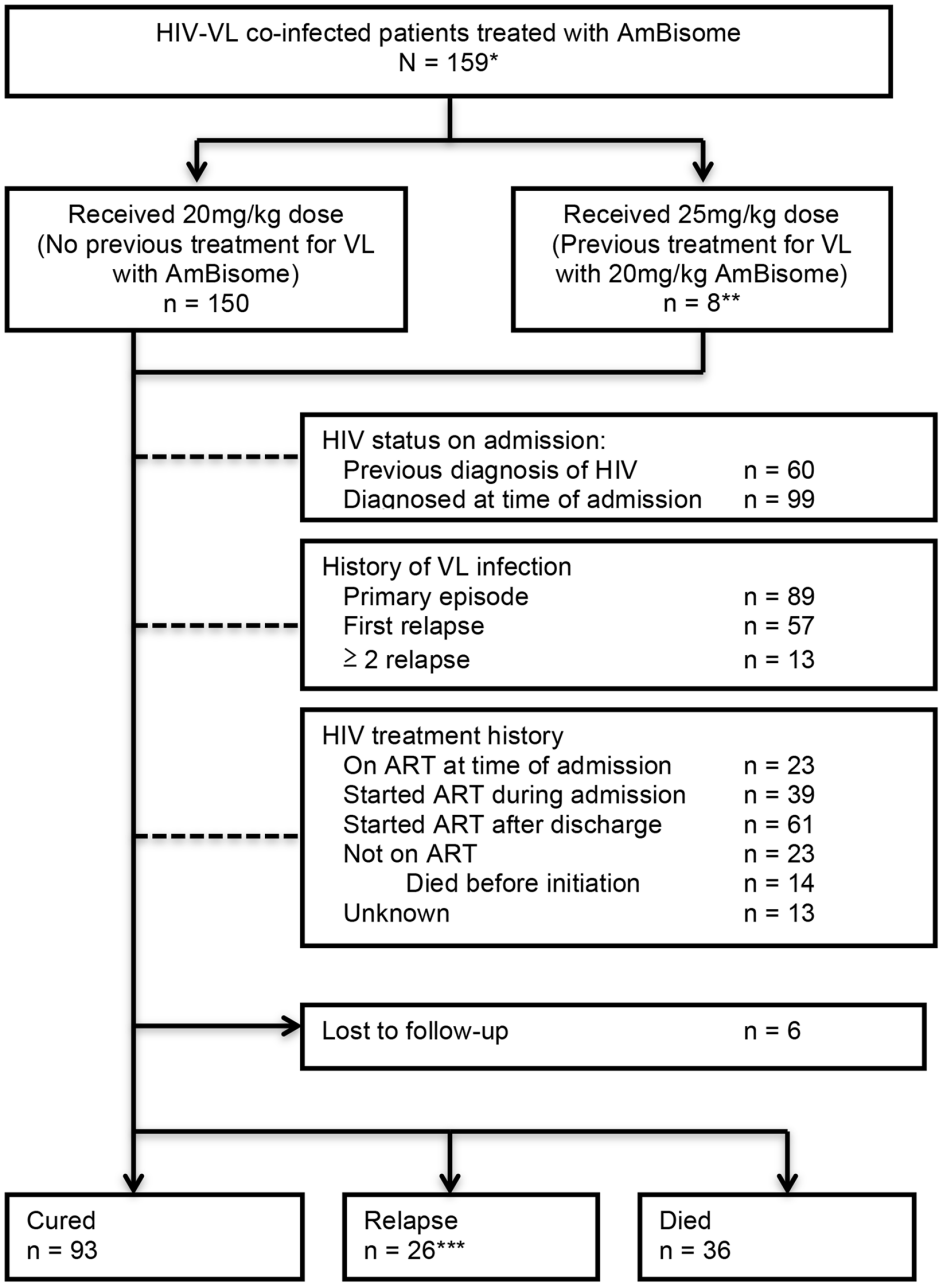
Patient characteristics and flow through VL treatment. Footnote: *A total of 161 patients were diagnosed with HIV-VL co-infection in the program. One HIV-VL patient died before any treatment was given, and another was diagnosed with HIV at an external facility 3 months after completion of treatment. ** 1 patient was treated with total dose of 15 mg/kg due to pre-existing renal failure (included in analysis). *** 2 patients who relapsed subsequently died. HIV – Human Immunodeficiency Virus, VL – Visceral Leishmaniasis, ART – Antiretroviral Therapy.

In collaboration with RMRI, MSF developed a comprehensive VL program that was fully integrated into the MoH facilities in Vaishali district, Bihar. This program encompassed two main activities: the running of an MSF-led inpatient unit within the district hospital, and the provision of logistic support for VL services to five Primary Health Centers (PHCs) within Vaishali district. From February 2007 until August 2012, 8,749 patients were treated for VL using 20 mg/kg liposomal amphotericin B as first-line therapy, as described in detail elsewhere [18]. Approximately 50% of these patients originated from Vaishali district, while the remainder travelled from adjacent districts. Over time, rising numbers of patients entered the program via referrals from neighbouring district hospitals. In particular, HIV treatment centers outside the district increasingly referred patients who had been diagnosed with HIV and were thought to have VL. During the 5-year study period, the VL program treated 159 HIV co-infected patients, of whom 60 were directly referred by outside HIV treatment centers (Figure 1).

Data collected for all patients diagnosed with VL included general demographic information, clinical history, hemoglobin level, height, weight, and malaria rapid diagnostic test result. The study also recorded information on ‘caste’, a form of social stratification used in India, using the following categories and definitions: scheduled caste and scheduled tribe (terms used for two groups of historically disadvantaged people recognized in the Constitution of India); other backward class (a collective term used by the government of India for castes that are educationally and socially disadvantaged but not specifically mentioned in the Constitution); and general category (not considered to be disadvantaged). The first three groups combined account for approximately 60% of India's population.

### Diagnosis of Visceral Leishmaniasis

All patients with a history of 2 weeks fever and clinical splenomegaly were considered suspect for VL. Diagnosis was confirmed with the rK39 rapid diagnostic test (DiaMed-IT LEISH; DiaMed AG, Cressier, Switzerland). Patients presenting with a history suggestive of relapse or with atypical clinical signs or negative diagnostic tests but a high index of suspicion of VL were referred to the RMRI for parasitological diagnosis through splenic or bone marrow biopsy. Of the 159 co-infected patients, 31 had the diagnosis of VL confirmed solely with rk39 serological testing, with the remainder through parasitological visualization on biopsy using established techniques [19].

### Diagnosis of HIV

At the start of the program, only patients with a history suggestive of possible HIV exposure were offered provider-initiated counseling and testing (PICT) for HIV. Indications for PICT included a history of relapse, a high-risk profession or being a migrant worker, but were otherwise not clearly defined. Patients self-reporting a previous diagnosis of HIV were counseled and re-tested, as were patients referred from external hospitals presenting with a provisional diagnosis of HIV-VL co-infection. However, a few patients treated for VL and discharged from the program subsequently re-presented with confirmed relapse, at which point they were tested and diagnosed with HIV infection. Therefore, the HIV testing policy was changed in March 2011, after which all patients aged ≥14 years were offered PICT for HIV.

HIV testing at the program facility was initially performed using two rapid diagnostic tests in parallel (SD Bioline HIV 1/2 and Determine-HIV 1/2), with patients testing positive referred to MoH testing facilities for further diagnosis as per local protocols using Combaids Advantage, TriLine and TriSpot RDTs. However, use of SD Bioline was stopped in December 2011, in keeping with WHO recommendations [20]. From that point onwards, patients testing positive with Determine-HIV 1/2 tests alone were referred. Discordant tests were confirmed with Western Blot.

### Treatment and follow-up

Patients with or without HIV who presented to the program for the first time were treated under the same protocol, using 20 mg/kg intravenous liposomal amphotericin B given in 4 doses over 4–10 days depending on the severity of illness. Patients considered to be in good clinical condition were treated on 4 consecutive days, while those requiring a longer period of inpatient observation received the 4 doses over 10 days. All patients diagnosed with a VL relapse and who had previously received the 20 mg/kg liposomal amphotericin B regimen at the program were treated with an increased dose (25 mg/kg) of liposomal amphotericin B following parasitological confirmation of relapse. All patients presenting with VL relapse were offered PICT, Patients were considered ‘initial cures’ once they completed a full course of VL treatment and showed clinical improvement, cessation of fever, reduction of spleen size and return of appetite at the time of discharge following WHO descriptions of treatment response [21].

Test of Cure (ToC) was not routinely performed, due to the risks associated with splenic puncture and in light of a previous study showing a cure rate of >98% at 6 months [22], Instead, splenic or bone marrow aspiration was reserved for confirmation of VL in all patients presenting with relapse, those with suspected initial treatment failure, and initially, for all HIV-VL co-infected patients. However, as neither relapses nor treatment failures occurred in any of the 55 HIV-VL patients treated during the first 6 months, [23], from that point onwards ToC was not routinely performed in this group.

Following completion of VL treatment and improvement of their general condition, patients with HIV–VL co-infection were offered antiretroviral treatment (ART) at either the RMRI or within Vaishali district hospital, using the national program-recommended regimen of stavudine, lamivudine and nevirapine. However, as a growing number of national program centers providing ART opened in Bihar over time, responsibility for initiation and maintenance of ART was transferred to these more local facilities, which helped create a more sustainable, patient-friendly treatment strategy. Communication between the VL treatment program and the ART treatment centers was maintained, and patients with suspected VL relapse were referred back to the MSF program for diagnosis and treatment. In addition, the centers shared information on ART patient adherence and CD4 counts, where available.

After successful completion of VL treatment, patients were asked to return to the MSF program for follow-up at 1, 3, 6, 12 and 24 months from the time of VL treatment initiation, They were also counseled at discharge regarding the high risk of relapse and the importance of adherence to ART. Secondary prophylaxis against VL, which is recommended elsewhere [8], [17], was not offered. The program made considerable effort to maintain long-term contact with patients though active telephone tracing and, in cases where contact through telephone tracing or ART treatment centers failed, through home visits.

### Data collection

All data were entered into a standard Microsoft Excel database; double data-entry was not done at the time of inputting. Regular database cleaning consisted of checks for inconsistencies, with reference to source documents where necessary. Although an epidemiologist ensured the database was well maintained and regularly audited the quality of data transfer, all records of co-infected patients were reviewed again immediately before the final analysis to ensure that data entered into the database was correct.

Nutritional status (Body Mass Index, BMI) was assessed using weight and height data for patients ≥19 years of age, while World Health Organization Anthro and Anthro Plus software (Geneva, Switzerland) was used to calculate a BMI-for-age Z-score for those aged ≥5–19 years and Weight for Height (W/H) Z-score for those 6 months to <5 years of age. For patients ≥19 years of age, severe acute malnutrition (SAM) and moderate acute malnutrition (MAM) were defined as BMI<16 and 16–<17, respectively. For patients aged ≥5–19 years, SAM was defined as BMI-for-age Z-score <−3 and MAM as <−2 but >−3 SD, while for patients aged 6 months–<5 years, SAM and MAM were defined as W/H Z-score <−3 and <−2 but >−3 SD, respectively.

### Data analysis

Statistical analysis of data was conducted using STATA version 11 (STATACorp LP, College Station, USA). For the final analysis all data were anonymised. Baseline characteristics for all co-infected patients were compared against VL patients not known to be HIV-positive and treated in the program over the same time period. Primary outcomes were time to death and time to relapse. Person-time at risk was calculated for each patient, starting from the date of VL treatment initiation up to the date of death, date of the last visit (for those lost to follow-up), or 31^st^ August 2013 (for everyone else). With relapse as outcome, follow-up time started with hospital discharge and ended at the date of (first) relapse for those with relapse. The cumulative incidence of mortality or relapse was estimated using Kaplan-Meier methods. A risk factor analysis was performed using multivariate Cox regression modelling. Variables considered for inclusion were age, sex, a history of VL, ART use and the following characteristics (all at the time of VL diagnosis): hemoglobin level, body mass index, spleen size, CD4 cell count, concurrent tuberculosis, and duration of illness (only in relapse analysis).

For those initiating ART, the overall change in CD4 count levels after diagnosis of HIV-VL co-infection was visualized using a nonparametric method called LOWESS smoothing (for locally weighted scatterplot smoothing, ‘lowess’ command in STATA). This provides a representative smooth curve through data using robust local regression.

In the mortality analysis, ART use was categorized as either 1) being on ART at the time of VL diagnosis, or 2) ART initiation after VL diagnosis, included as a time-varying covariate. With relapse as outcome, ART use was categorized as 1) being on ART at the time of VL diagnosis; 2) ART initiation during admission: 3) ART initiation sometime after admission, included as a time-varying covariate. In the main analysis, variables associated with the outcome with a *P*-value <0.05 in univariate analysis were included in multivariate analysis. The model was reduced by backward stepwise elimination until all variables had a *P*-value <0.05. In secondary analysis, ART use was forced in the model.

In the main analysis, multiple imputation was used for missing data [24]. In sensitivity analysis 1, we used the missing indicator method (whereby for a specific predictor a separate category is generated for missing data). Continuous co-variables were categorized in the main analysis but included as continuous variables in sensitivity analysis 2, with the functional form determined using the multivariable fractional polynomial (mfp) models command in STATA. Several other secondary and sensitivity analyses were also conducted, including the removal of patients with incomplete information on ART use in models involving this parameter.

In addition, the cumulative incidence of relapse was recalculated to allow for the presence of competing risks (death precluding the occurrence of relapse), since in this case standard survival methods can lead to biased estimates [25], [26]. The proportional hazard assumption was assessed graphically and tested formally using Schoenfeld residuals. Co-linearity was evaluated by calculating the variance inflation factors. The level of significance was set at *P*<0.05.

### Ethics statement

This analysis met the Médecins Sans Frontières Institutional Ethics Review Committee criteria for a study involving the analysis of routinely collected program data. Although AmBisome is a new treatment in the Indian setting, it is a recognized treatment for VL; moreover, the program was run in coordination with the State Health Society through a memorandum of understanding, which is the usual procedure for NGOs operating in this context. The HIV-VL clinical treatment guideline had been reviewed and approved by the RMRI Institutional Ethics Committee. All electronic data were analyzed anonymously.

## Results

A total of 159 HIV/VL co-infected patients were treated for VL during the 5-year MSF-supported program (July 2007 to August 2012). Of these, 150 (94.3%) were treated with 20 mg/kg liposomal amphotericin B, while 8 (5%) were treated with a 25 mg/kg regimen. All patients completed treatment with no discontinuations or serious adverse events, and no deaths were associated with liposomal amphotericin B treatment. Four patients were lost to follow up within one month after VL diagnosis, one at six months and one at twelve months. The maximum length of follow-up was 5.6 years following completion of VL treatment, with a mean of 2 and median of 1.2 years.

### Patient characteristics

The demographic and baseline clinical characteristics of the 159 co-infected patients compared to the remaining treated cohort of 8,590 patients not known to be HIV-positive are shown in Table 1. The Relative Risk (RR) of being HIV-positive was 3.7 times higher for males than for females, while the RR of being HIV-positive was 25 times lower (0.04, 0.02–0.07) for patients <25 years of age compared to those aged 25–<55 years. The mean ±SD age was 36.6±10.4 years (range 7–70); 145 (91.2%) patients were aged 25–55 years, of whom 64 (44.1%) were aged 35–45 (Table 1). The RR of being co-infected with HIV and of a General Category caste was 2.2 (95% Confidence Interval 1.6–3.1) times higher than compared to being of an Other Backward Class or Scheduled Caste. Compared to patients not known to be HIV-positive, those with HIV-VL co-infection tended to have a lower hemoglobin (<6 g/dl) on admission (RR 1.7, 1.02–2.7, p = 0.04) and a greater degree of splenomegaly >6 cm (RR 2.1, 1.6–2.9, p<0.001).

**Table 1:** Demographic and clinical admission characteristics of HIV-positive patients treated for VL and patients not known to be HIV-positive.

	HIV-VL N = 159		Remaining cohort N = 8590			
Variable*	N	%	N	%	RR (95%CI)	P value
**Sex (n = 8749)**						
Male	132	83.0	4868	56.7	3.7 (2.4–5.5)	**<0.001**
Female	27	17.0	3722	43.3	1	
**Age group (years) (n = 8749)**						
<14	5	3.1	3686	42.9	0.02 (0.01–0.05)	**<0.001**
14–<25	4	2.5	1539	17.9	0.04 (0.02–0.1)	**<0.001**
25–<35	49	30.8	1193	13.9	0.6 (0.4–0.9)	**0.01**
35–<45	64	40.3	945	11.0	1	
45–<55	27	17.0	596	6.9	0.7 (0.4–1.1)	**0.09**
≥55	10	6.3	631	7.3	0.3 (0.1–0.5)	**<0.001**
**Caste (n = 8695)**						
Scheduled Caste	22	14.1	2502	29.3	0.3 (0.8–1.9)	**<0.001**
Other Backward Class	89	57.1	4722	55.3	0.6 (0.4–0.8)	**0.001**
General Category	45	28.8	1315	15.4	1	
**Hemoglobin (g/dl) (n = 8715)**						
<6	22	14.2	1112	13.0	1.2 (0.8–1.9)	**0.425**
6–8	58	37.4	2841	33.2	1.3 (0.9–1.8)	**0.199**
>8	75	48.4	4604	53.8	1	
**CD4 count, cells/uL (n = 122)** **						
<100	56	45.9	-	-	-	-
100–199	36	29.5	-	-	-	-
200–349	23	18.9	-	-	-	-
≥350	7	5.7	-	-	-	-
**Time from symptoms onset to diagnosis (n = 8738)**						
>8 weeks	45	29.4	1459	17.0	1.7 (1.02–2.7)	**0.04**
>4–8 weeks	41	26.8	2108	24.6	1.1 (0.6–1.8)	0.813
>2–4 weeks	43	28.1	3706	43.2	0.6 (0.4–1.05)	0.07
≤2 weeks	24	15.7	1312	15.3	1	
**History of previous treatment for VL (n = 8749)**						
Yes	70	40.0	325	3.8	16.6 (12.4–22.4)	**<0.001**
No	89	56.0	8265	96.2	1	
**Spleen size, cm (n = 8741)**						
>6	82	52.9	2936	34.2	2.2(1.3–3.7)	**0.004**
3–6	57	36.8	4395	55.2	1.02(0.6–1.8)	0.952
<3	16	10.3	1255	14.6	1	
**Nutrition status (n = 7252)**						
SAM	37	23.9	1270	17.9	1.4(0.95–2.0)	**0.095**
MAM	30	19.4	1623	22.9	0.9(0.6–1.3)	0.56
Normal	88	56.8	4204	59.2	1	

* Where n<8749, this is due to missing data.

** Window 6 months prior to VL treatment until 6 weeks after.

*VL – Visceral Leishmaniasis; HIV – Human Immunodeficiency Virus; SAM – Severe Acute Malnutrition; MAM – Moderate Acute Malnutrition; RR – Relative Risk; CI – Confidence Interval*.

There was no significant difference between the global nutritional status of patients known to be HIV-positive and the remainder of the cohort (43.3% vs 40.8% globally malnourished respectively, RR (95%CI) = 1.1 (0.8–1.5), p = 0.54), nor was there a significant difference in the prevalence of severe acute malnutrition (SAM) between patients known to be HIV-positive and the remainder of the cohort – 23.9% vs 17.9%, respectively, RR = 1.4 (95%CI 0.95–2.0, p = 0.095).

However, the RR of presenting with a relapse of VL was particularly high – the odds of being HIV-positive and having previously experienced a single or multiple episodes of VL prior to admission was 16.6 times higher than in the overall cohort (95% CI 12.4–22.4; p<0.001).

Of the 159 co-infected patients, 60 (37.7%) had been diagnosed with HIV prior to attending the MSF program, of which less than half (23) were receiving ART at time of diagnosis of VL. The remaining 99 (62.3%) patients were diagnosed with HIV at the time of VL diagnosis. 122 (76.7%) of patients had CD4 counts recorded between 6 months before or one month following treatment for VL, with a mean count of 122 cells/uL, and a median of 111 (IQR 59-193). Nearly half (n = 56, 46%) had counts <100 cells/uL. Patients already on ART at time of treatment for VL had a median CD4 count of 188 cells/ul (IQR 54-164), while patients not on ART had a median CD4 count of 101 cells/ul (IQR 75-234). Patients presenting with primary or a previous episode of VL had a median CD4 counts of 108 cells/uL (IQR 57-163) and 113 cells/uL (IQR 61-216) respectively. A total of 9 (5.7%) patients were suffering from tuberculosis in addition to HIV-VL co-infection.

### Survival

**Figure 2:**
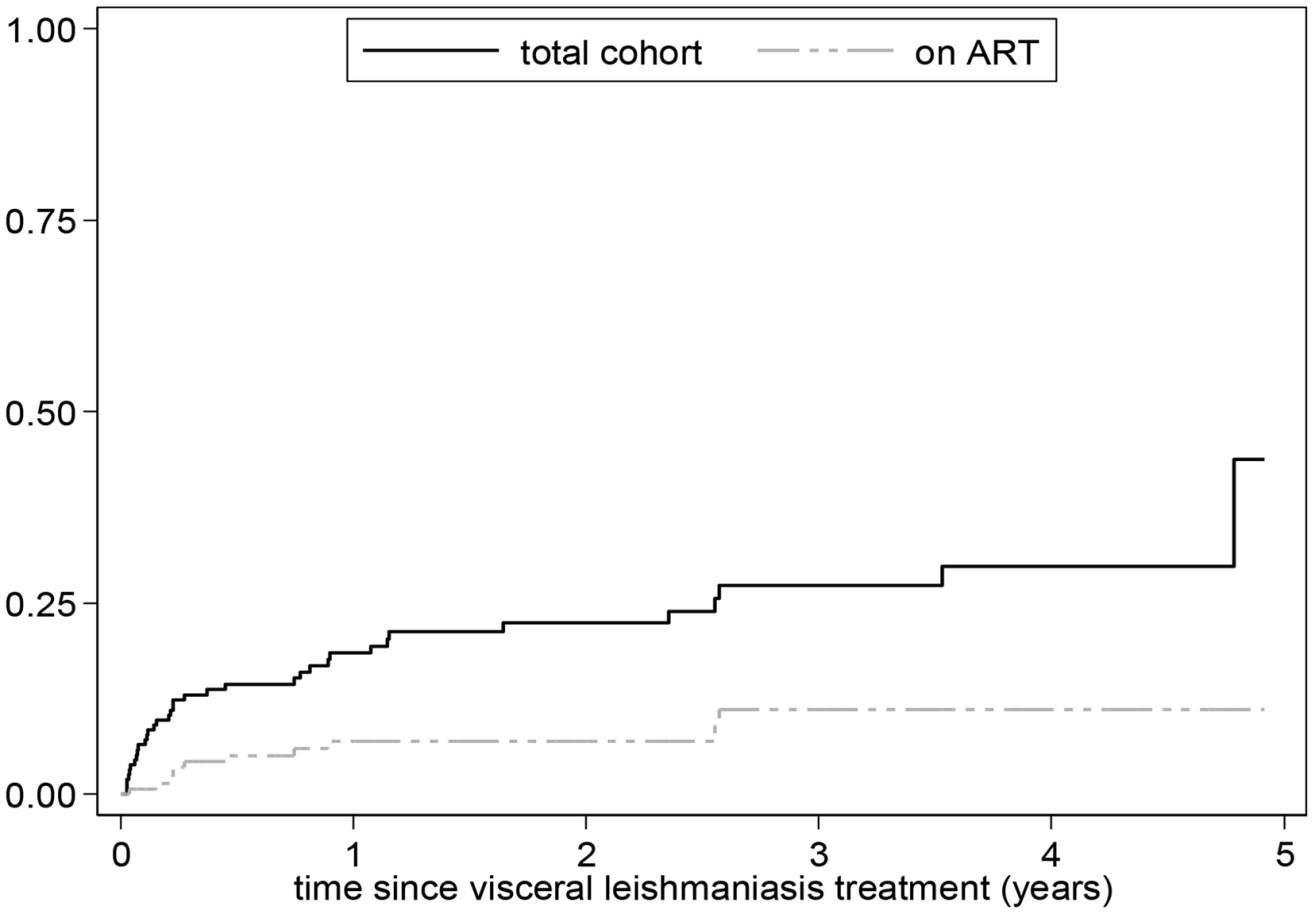
Censored Kaplan Meier curve showing the cumulative hazard of death over time following discharge.

A total of 36 co-infected patients died, including six who died shortly after admission. Death occurred at a median of 11 weeks (Inter-Quartile Range, IQR 4-51) after starting VL treatment. The estimated mortality risk was 14.3% at six months, 22.4% at two years and 29.7% at four years after diagnosis (Figure 2). In univariate analysis, a low BMI (<16 kg/m^2^), low hemoglobin (<7 g/dL) at admission, and concurrent tuberculosis were each associated with an increased risk of mortality (Table 2). Use of ART was associated with a decreased risk.

**Table 2:** Univariate analysis to determine risk factors for mortality in VL/HIV co-infection.

Variable	Crude HR (95% CI)	*P*-value
Male sex	1.16 (0.45–2.98)	0.76
Age >40 years	1.60 (0.81–3.18)	0.17
TB diagnosis*	4.24 (1.75–10.24)	**0.001**
History of VL*	0.81 (0.41–1.59)	0.53
Spleen size >10 cm*	0.86 (0.30–2.49)	0.79
BMI<16 kg/m^2^ *	2.06 (1.04–4.10)	**0.039**
Hemoglobin <7 g/dL*	2.55 (1.31–4.99)	**0.006**
CD4 count <50 cells/µL*	1.30 (0.60–2.80)	0.51
On ART at VL diagnosis^a^	0.26 (0.07–0.93)	0.038
ART started after VL diagnosis^a^	0.29 (0.12–0.68)	0.004

* at time of VL diagnosis;

a Compared to those never started on ART.

*VL – Visceral Leishmaniasis; HIV – Human Immunodeficiency Virus; BMI – Body Mass Index; HR – Hazard Ratio; CI – Confidence Interval; ART –Antiretroviral Therapy, TB – tuberculosis*.

In multivariate analysis, concurrent tuberculosis and low hemoglobin were independent risk factors. This remained true when ART use was included the model. ART use was associated with a 64–66% reduced risk of mortality, but the effect only reached statistical significance when ART was initiated after VL diagnosis (Table 3). Minor changes in the estimates were observed in the sensitivity analyses.

**Table 3:** Independent risk factors for mortality in VL/HIV co-infection.

	Adjusted HR (95% CI)	*P*	Adjusted HR (95% CI)	*P*
Main analysis	Stepwise model		Including ART	
Tuberculosis*	3.92 (1.62–9.50)	0.002	3.40 (1.39–8.33)	0.007
Hemoglobin <7 g/dL*	2.44 (1.25–4.78)	0.009	2.16 (1.09–4.28)	0.027
On ART at VL diagnosis	-		0.34 (0.09–1.24)	0.10
ART initiation after VL	-		0.36 (0.15–0.85)	0.021
**Sensitivity analysis 1**				
Tuberculosis*	3.9 (1.6–9.5)	0.002	3.5 (1.4–8.5)	0.006
Hemoglobin <7 g/dL*	2.4 (1.2–4.8)	0.010	2.2 (1.1–4.3)	0.026
On ART at VL diagnosis	-		0.35 (0.10–1.28)	0.11
ART initiation after VL	-		0.38 (0.16–0.90)	0.028
**Sensitivity analysis 2** 1				
Age (per 5 years increase)*	1.26 (1.09–1.46)	0.002	1.28 (1.19–1.48)	0.001
BMI (per 1 kg/m2 increase)*	0.81 (0.69–0.95)	0.009	0.82 (0.69–0.98)	0.030
Hb (per 1 g/dL increase)*	0.79 (0.66–0.95)	0.011	0.82 (0.68–0.98)	0.029
On ART at VL diagnosis^a^	-		0.34 (0.10–1.51)	0.17
ART initiation after VL^a^	-		0.33 (0.14–0.80)	0.014

* at time of VL diagnosis;

a Compared to those never started on ART.

1 Tuberculosis not retained, but borderline significant (adjusted HR: 2.5 (95% CI 1.0–6.2); *P* 0.053 in stepwise model).

Sensitivity analysis 1: alternative strategy to account for missing data.

Sensitivity analysis 2: continuous co-variates entered in original form (no categorization).

*VL – Visceral Leishmaniasis; HIV – Human Immunodeficiency Virus; BMI – Body Mass Index; HR – Hazard Ratio; CI – Confidence Interval; ART –Antiretroviral Therapy*.

### VL treatment response

**Figure 3:**
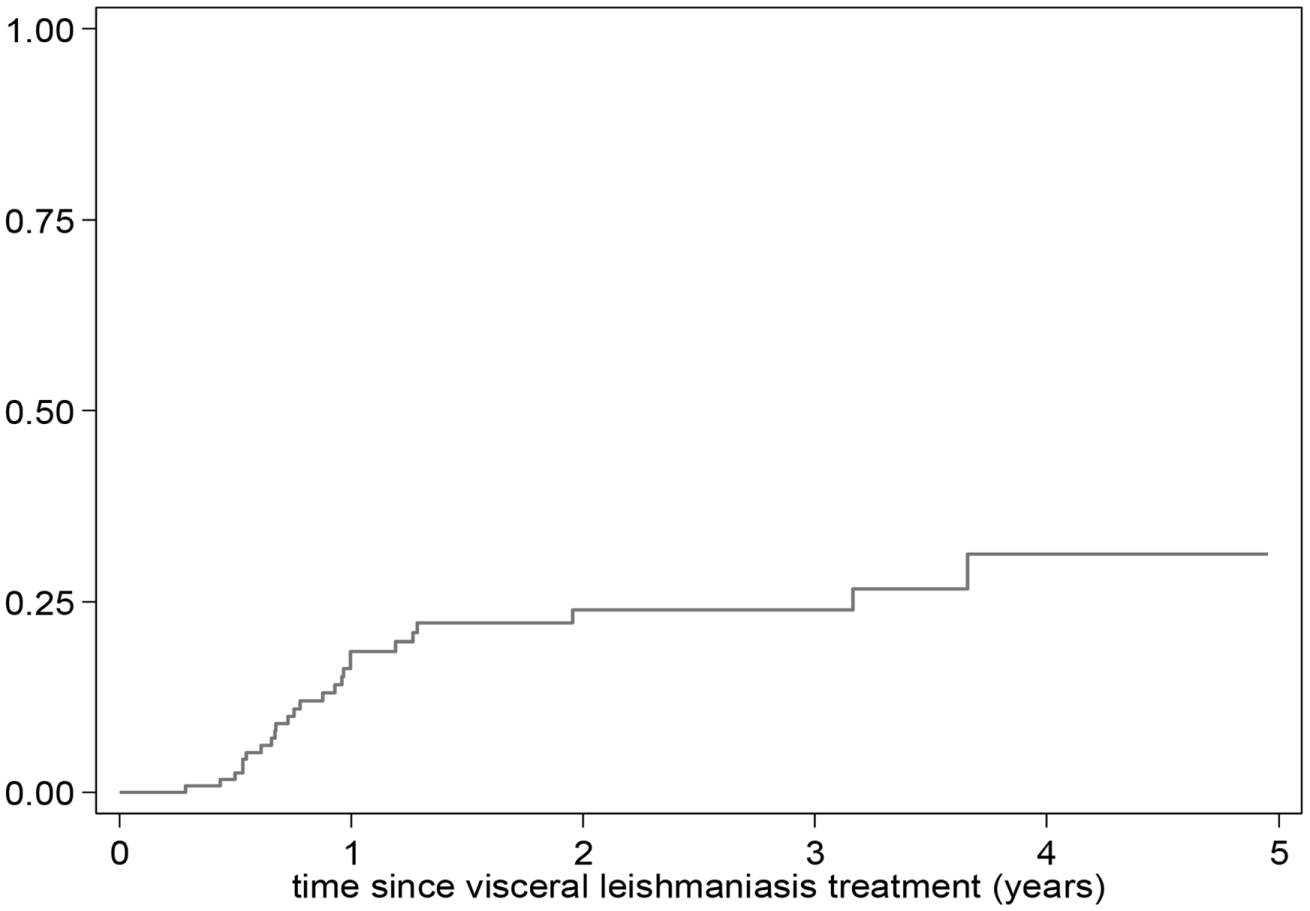
Censored Kaplan Meier curve showing the cumulative hazard of relapse over time following discharge.

Although all patients completed treatment and showed clinical improvement, six patients died following a period of prolonged admission, likely due to multiple contributing factors. From the remainder, there were no documented cases of treatment failure based on treatment response. Among the 153 patients discharged from the hospital, a total of 26 cases of VL relapse were diagnosed during follow-up, occurring at median of 10 months (IQR 7-14) after discharge. The estimated risk of relapse was 1.6% at six months after discharge, but subsequently increased to 18.5% at one year and 23.8% at two years (Figure 3). Four years following VL treatment the risk was 31.2%.

In univariate analysis, age >40 years was associated with an increased risk of relapse (Table 4). This effect showed borderline significance when ART use was included in the model. The association of ART use with a reduced risk of relapse (75% reduction) was statistically significant only for ART initiation during admission.

**Table 4:** Risk factors for relapse in VL/HIV co-infection in univariate analysis.

Variable	Crude HR (95% CI)	*P*-value
Male sex	1.32 (0.39–4.41)	0.65
Age >40 years	2.29 (1.02–5.12)	**0.043**
Duration of illness >4 weeks	0.69 (0.30–1.60)	0.39
TB diagnosis*	1.99 (0.47–8.46)	0.35
History of VL*	0.81 (0.36–1.82)	0.61
Spleen size >10 cm*	1.36 (0.41–4.52)	0.61
BMI<16 kg/m^2^ *	0.78 (0.28–2.17)	0.63
Hemoglobin <7 g/dL*	0.61 (0.21–1.86)	0.38
CD4 count <50 cells/µL*	0.73 (0.24–2.26)	0.58
On ART at VL diagnosis^a^	0.55 (0.15–2.09)	0.38
ART initiation during admission^a^	0.24 (0.07–0.81)	0.022
ART initiation after discharge^a^	0.51 (0.17–1.51)	0.22

* at the time of VL diagnosis;

a Compared to those never started on ART.

*VL – Visceral Leishmaniasis; HIV – Human Immunodeficiency Virus; BMI – Body Mass Index; HR – Hazard Ratio; CI – Confidence Interval; ART – combination Antiretroviral Therapy, TB – tuberculosis*.

**Figure 4:**
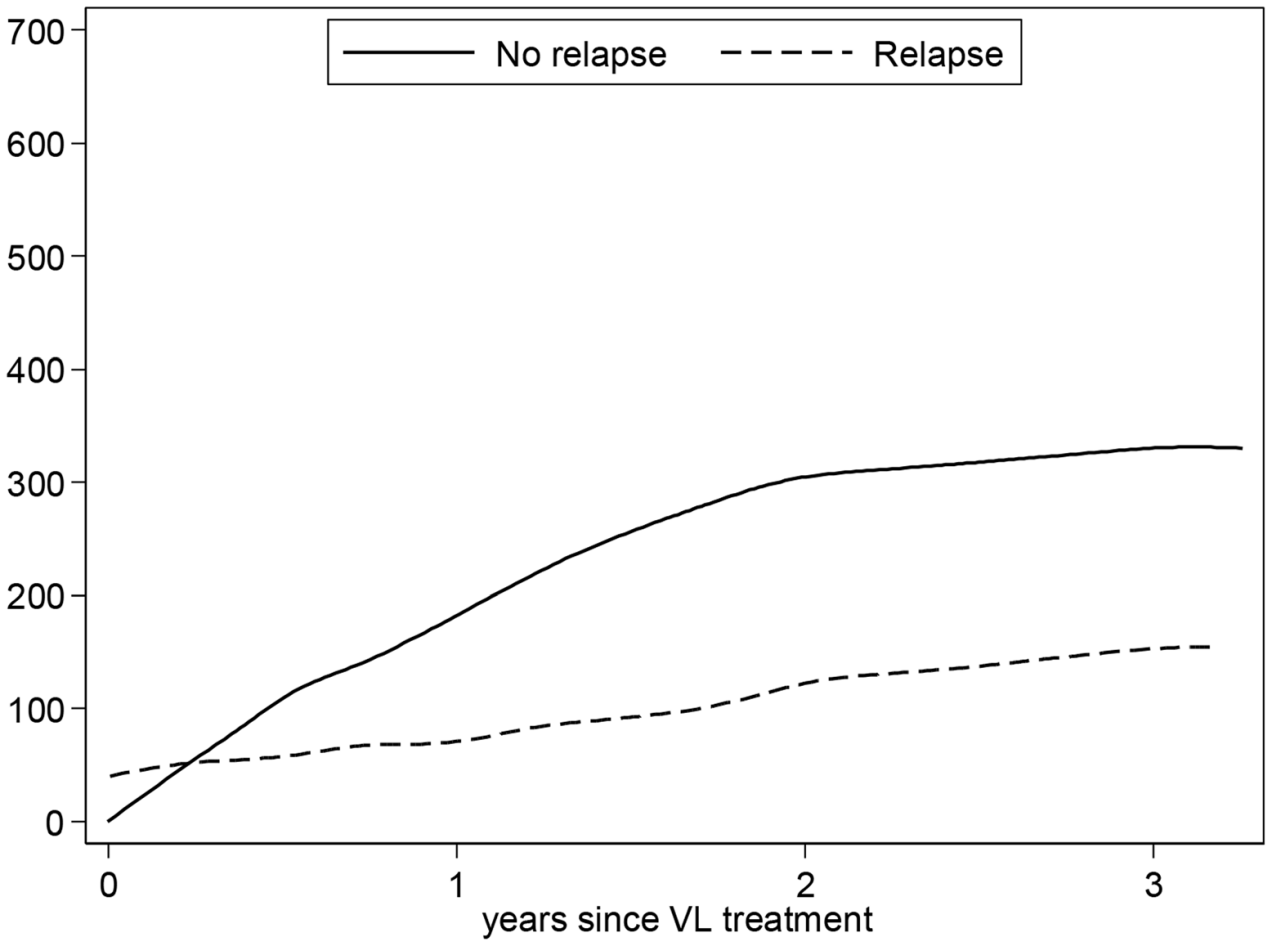
Evolution of CD4 count* following treatment for VL in patients who relapsed compared to those who did not. Footnote: *Timeline restricted to 3 years as subsequent data points were limited.

We also examined whether CD4 cell count recovery following VL treatment was associated with the risk of relapse. As shown in Figure 4, CD4 recovery was blunted in patients who subsequently relapsed compared to those who remained relapse-free. The median CD4 count of patients who relapse and did not subsequently relapse following treatment was 95 cells/uL, (IQR 63-163) versus 112 cells/uL, (IQR 57-206), respectively. Of the 26 patients who subsequently relapsed, 16 had a CD4 count recorded around the time of relapse, with a median count of 137 cells/uL (IQR 80-255).

Accounting for competing risks, the estimated risk of relapse decreased to 16.1% at one year, 20.4% by two years and 25.9% by four years. The main findings remained unchanged by the other sensitivity analyses (Table 5).

**Table 5:** Independent risk factors for relapse in VL/HIV co-infection.

	Adjusted HR (95% CI)	*P*	Adjusted HR (95% CI)	*P*
Main analysis	Stepwise model		Including ART	
Age >40 years	2.29 (1.02–5.12)	0.043	2.20 (0.98–4.95)	0.055
On ART at VL diagnosis^a^	-		0.54 (0.14–2.04)	0.36
ART initiation during admission^a^	-		0.25 (0.07–0.84)	0.026
ART initiation after discharge^a^			0.52 (0.18–1.56)	0.25
**Sensitivity analysis 2** 1				
Age (per 5 years increase)	1.24 (1.04–1.48)	0.018	1.22 (1.03–1.45)	0.021
On ART at VL diagnosis^a^	-		0.52 (0.14–1.98)	0.34
ART initiation during admission^a^			0.24 (0.07–0.80)	0.020
ART initiation after discharge^a^	-		0.50 (0.17–1.47)	0.20

a Compared to those never started on ART.

1 Sensitivity analysis 1 (not shown) yielded exactly the same results as the main analysis since there were no missing data for the variables included.

Sensitivity analysis 2: continuous co-variates entered in original form (no categorization).

*VL – Visceral Leishmaniasis; HIV – Human Immunodeficiency Virus; HR – Hazard Ratio; CI – Confidence Interval; ART –Antiretroviral Therapy*.

## Discussion

This study describes the admission characteristics and long-term VL treatment outcomes for the largest cohort of HIV-VL co-infected patients from the Indian subcontinent, with a longer follow-up period and lower rate of loss-to-follow-up than any report published to date. As in previous studies, our data show high mortality in these patients, particularly in the early period following diagnosis, and a high VL relapse rate, findings which underscore the crucial importance of early diagnosis and intervention for both diseases.

Early initiation of ART had a clear impact on reducing mortality and relapse, and should therefore be considered a key intervention in the management of these patients. In agreement with our earlier estimates of 2-year outcomes for the first 55 HIV-VL patients in this cohort [23], outcomes for this much larger number of co-infected patients were substantially worse than for VL patients not known to be HIV-positive: after receiving the same VL treatment in the same setting, estimated all-cause mortality and relapse rates at 15 months for patients not known to be HIV positive were 2.8% and 1.2% respectively [18], compared to 18.1% and 16.1% at 12 months respectively for co-infected patients reported in this study.

Concurrent infection with tuberculosis and hemoglobin <7 g/dl were independently associated with mortality. In terms of protective factors, ART initiated immediately following VL treatment was associated with a 64–66% reduced risk of mortality (p<0.05). Similar reductions in mortality risk for co-infected patients adherent to ART have been reported in other studies [8]. Our data suggested that ART use *prior to* VL diagnosis may also be associated with reduced mortality, but this association did not reach statistical significance. This lack of a demonstrated effect of prior ART could be due simply to the relatively small number of patients, or could reflect the possibility that patients already on ART at time of VL diagnosis may have been experiencing ART treatment failure or more advanced disease – or conversely that those with more favorable responses to ART may be at much lower risk of developing VL and therefore never enroll in the program, leading to an underestimation of the effect of ART. Notably, baseline CD4 counts around the time of VL diagnosis were typically very low in our cohort (mean baseline CD4 count 122 cells/ul).

In terms of VL relapse risk, we did not detect clear demographic associations, in agreement with results from a recent systematic review looking at predictors of VL relapse in HIV-positive patients [27]. However, initiation of ART immediately following VL treatment was associated with a significant reduced risk of relapse (although not if initiated either before or long after VL treatment), while a history of one or more previous VL episodes at time of treatment was not. These results differ from those seen in the meta-analysis, which concluded that ART did not appear to reduce the risk of relapse whereas a previous history of VL was predictive of relapse. However, other than one from Ethiopia, all studies included in this meta-analysis were conducted in Europe, the majority with small sample sizes and limited follow-up periods.

The Ethiopian study alone involved VL caused by *L. donovani*, as in our Indian setting. Infection with *L. donovani* has different clinical implications compared to *L. infantum*, the causal agent in most European and Latin American VL cases, and therefore more relevant to HIV/VL management in India. The study found that ART was partially protective against VL relapse, while a baseline CD4 count of <100 cells/uL and a history of two or more relapses were associated with increased risk of relapse [28], however findings may have been biased by the high proportion of patients not receiving ART who were lost to follow up. In contrast, predicting relapses in India appears more complex as there did not appear to be any effect of either baseline CD4 count or of previous VL history.

### Limitations of this study

This study has a number of limitations. Primarily, although admission and VL treatment data had relatively few missing values, data from the HIV management perspective was incomplete; as follow-up periods extended past 3 years, the number of available CD4 counts decreased, which prevented further accurate modeling. Second, a larger sample size may have yielded more precise estimates for both risk factors and measures of outcomes. Third, the prevalence of HIV-VL co-infection cannot be estimated from this study, since all patients were not systematically screened for HIV, and it is likely that a substantial number of co-infected patients were missed in the overall treated cohort.

Another limitation was that we considered all-cause mortality in the analysis, therefore excluding the possibility that death may have occurred due to other causes unrelated to HIV-VL. A further weakness is that the analysis included 5% (n = 8) of the patients who received a 5 mg/kg higher dose of AmBisome than the remainder. Lastly, although no initial treatment failures were seen in patients discharged from the program, it is likely that the routine use of ToC would have identified treatment failures that were missed clinically. It is unclear what the value of partial response patterns (eg partial but not complete regression of splenomegaly) is in determining true treatment response, particularly in co-infected patients. An Ethiopian study with systematic ToC found 32% parasitological failure in co-infected patients after treatment with 30 mg/kg liposomal amphotericin B despite good clinical response [29].

Co-infected patients show decreased cellular and humoral response to *Leishmania* parasites and are considered difficult to achieve a definitive cure from VL. As such, suspicion of relapse is more challenging in co-infected patients, since these patients often have persistent haematological abnormalities and residual hepato-splenomegaly at the end of treatment. Indeed, worsening of these abnormalities in the absence of fever may itself represent a new episode of VL, and as such it is plausible that there was under-reporting of relapse cases due to the importance given to fever in the routine diagnosis of symptomatic VL.

### Implications for patient care and national VL programs

The findings from this cohort analysis have a number of implications for improving the outcome of HIV-VL co-infected in India. Recent studies in the Indian subcontinent have recommended increasing the routine follow-up period after VL treatment from 6 months to 1 year [30]–[32]. However, for HIV-VL co-infected patients, it appears that the risk of relapse is greatest within 18 months following treatment, suggesting that routine follow-up should be extended even further for co-infected patients. Furthermore, if secondary prophylaxis is to be initiated, this period might be the most effective phase for its use.

Achieving longer follow-up without loss of many patients will/would require some changes to current practice, since maintaining long-term contact with patients who complete treatment is not integrated into existing VL programs, and without an existing framework, requires significant effort and resources. Similarly, there are no established mechanisms for sharing information about co-infected patients between the vertical VL and HIV programs in India. If such mechanisms were developed, they could facilitate more robust longer-term patient management, as has been seen in other co-infections, such as HIV/TB.

Although routine PICT for VL patients is recommended by WHO in areas where HIV counseling and access to ART are available [17], this service is lacking across the majority of endemic areas in India and is not included in the national VL program guidelines. Conversely, screening for VL in HIV-infected patients who have spent a significant amount of time in VL endemic areas is not mentioned by existing National AIDS Control Organisation (NACO) guidelines. We suggest that directives encouraging early diagnosis of co-infection are crucial as a means of reducing the high early levels of mortality observed in this study.

NACO guidelines recommend initiation of ART in all patients with clinical stage IV disease irrespective of CD4 count. However this recommendation refers to WHO guidelines, which identify ‘atypical disseminated visceral leishmaniasis’ as a stage IV defining opportunistic infection [33], rather than simply ‘visceral leishmaniasis’. This leads to confusion in the field when making decisions to start ART in co-infected patients, considering the WHO expert committee on VL clearly identifies HIV-VL co-infection as an AIDS defining illness [1]. Simultaneously, in the absence of national guidelines, maintaining consistent health messaging between parallel programs for HIV and VL is challenging. Reported non-adherence to ART regimens in India varies considerably, from 14%–86% [34]; in this study, 23 (14.9%) of patients either chose to discontinue, died prior to starting or did not start ART despite being referred to appropriate care providers. The provision of field-based guidance and training for the management of HIV-VL co-infection, as already exists for HIV-TB co-infection [35], [36], could be of great benefit in raising health provider awareness and improving management of these patients.

### Treatment challenges

This study suggests that 20–25 mg/kg liposomal amphotericin B is a well-tolerated and relatively effective treatment for HIV-VL co-infection in the Indian setting. However, these patients have a high risk of relapse, and clearly, repeated treatment with mono-therapy in cases of relapse may not be ideal as it may contribute to decreased drug susceptibility in the parasite [37]. Mechanisms for resistance to amphotericin B in clinical isolates of *L. donovani* have already been described [38], and decreased efficacy observed in co-infected patients after several treatment cycles [39], [40]. Additionally, unresponsiveness to liposomal amphotericin B seemed to develop rapidly in co-infected patients In Ethiopia where parasitological failure rates were 16% in primary HIV-VL, and 57% in relapse HIV-VL previously treated with AmBisome [29]. However, to date no parasite strains resistant to liposomal amphotericin B have been found, suggesting host-related factors may play a more important role in treatment unresponsiveness than parasite resistance. Although higher dose combination therapy has been recommended in cases of multiple VL relapse in co-infected patients [8] and has been successfully used in India [41], the use of such combinations needs to be further evaluated in the Indian subcontinent for all HIV-VL co-infected patients.

Within the field of TB-HIV co-infection, over the last 15 years there have been a number of observational studies conducted to understand the effect of ART on TB mortality, and the effect of timing of ART initiation. Combined, these allowed a clearer picture to emerge, which in turn contributed to the design of several clinical trials on the subject. In the absence of other studies from the Indian subcontinent within the field of HIV-VL co-infection, the data from this program constitutes a clear step forward, however highlights the need for additional studies to consolidate the evidence base and allow triangulation of different study findings.

Like patients with Post Kala-Azar Dermal Leishmaniasis (PKDL), HIV-VL patients harbor chronic infection, often have very high parasite loads and are therefore potential long-standing reservoirs for VL transmission. The role of asymptomatic VL infection has not yet been definitively established [42], however it is likely that an increase in HIV prevalence in endemic areas will lead to an associated increase in symptomatic VL infections. As such the importance of early identification, appropriate treatment, multidisciplinary management and follow-up of HIV-VL co-infected patients should be considered a public health priority if the goal of VL elimination is to be realized [43].

## Supporting Information

Checklist S1 STROBE checklist.
(DOC)
